# Astrocytes in Multiple Sclerosis: Getting to the Core

**DOI:** 10.3390/ijms27146520

**Published:** 2026-07-22

**Authors:** Rachel A. Tinkey, Brandon C. Smith, Jessica L. Williams

**Affiliations:** Department of Neurosciences, Cleveland Clinic Research, Cleveland Clinic, 9500 Euclid Avenue/NC30, Cleveland, OH 44195, USA

**Keywords:** multiple sclerosis, experimental autoimmune encephalomyelitis, astrocytes, neuroinflammation, chronic active lesions, demyelination, BATF2

## Abstract

Astrocytes are increasingly recognized as key regulators of multiple sclerosis (MS) pathogenesis, known to actively influence neuroinflammation, blood–brain barrier integrity, leukocyte recruitment, glial scar formation, and tissue repair. Evidence from both MS and its animal models demonstrates substantial astrocyte heterogeneity, exerting either pathogenic or neuroprotective functions depending on the local microenvironment. In this review, we discuss the diverse roles of astrocytes in MS, with particular focus on signaling pathways that govern astrocyte responses during chronic neuroinflammation. We further explore the growing evidence that astrocytes within chronic active lesion cores are not merely passive structural cells, but instead may be involved in immune regulation, survival signaling, and tissue maintenance. Collectively, these findings support a revised view of astrocytes as regulators of lesion dynamics and CNS homeostasis during MS. A deeper understanding of astrocyte heterogeneity and the signaling networks that control astrocyte function may reveal novel therapeutic opportunities to limit neurodegeneration and disease progression.

## 1. Introduction

Multiple sclerosis (MS) is a chronic disorder affecting the central nervous system (CNS) characterized by inflammatory demyelination and subsequent axonal injury, which can manifest clinically as sensory and motor deficits [1,2,3]. Most patients are diagnosed with relapsing–remitting MS (RRMS), in which episodes of recurrent neurological dysfunction are interspersed with periods of partial or complete remission. Initially, RRMS was viewed as being primarily driven by neuroinflammation; however, clinical studies have demonstrated that disability can accumulate independently of overt relapses, often referred to as progression independent of relapse activity (PIRA) or “silent progression”. These observations, together with imaging and biomarker studies, support a role for neurodegenerative mechanisms, in addition to inflammation, during early stages of disease and represent major drivers of long-term clinical worsening [4,5,6].

Historically, approximately half of RRMS patients were reported to convert to secondary progressive MS (SPMS) within 20 years of initial symptoms, while a smaller proportion, roughly 15%, present with primary progressive MS (PPMS), in which neurological decline is continuous from onset [7,8,9,10,11,12]. Importantly, these estimates were derived largely from untreated or pre-disease-modifying therapy (DMT) cohorts. More recent studies indicate that DMTs have delayed or, in some cases, reduced the risk of transition to SPMS; however, PIRA remains common even among treated patients [13,14,15]. Further, most currently available MS therapies are immunomodulatory and are generally effective at reducing inflammatory activity and relapses in RRMS; however, nearly all have reduced efficacy in patients with SPMS and PPMS and do not prevent progressive neurodegeneration in the absence of relapses [16,17,18,19,20].

Moreover, patients with MS exhibit widespread structural and pathological abnormalities, including changes in the normal-appearing white matter, cortical demyelination, and progressive brain atrophy [21,22,23,24,25,26]. Additional pathological hallmarks of MS include focal demyelinating lesions that are classified into several subsets including active, chronic active, and inactive lesions. While most lesion subtypes transition between these classifications, newly formed lesions are most commonly active [3,27,28,29]. Notably, active lesions contain a significant number of infiltrating peripheral immune cells, accompanied by activated resident glia, creating a highly inflammatory microenvironment [27,29,30]. Secretion of immune molecules, including cytokines, by these immune cells and glia contributes to the death and phagocytosis of oligodendrocytes (OLs) and their associated myelin, leading to demyelination and, in severe circumstances, axonal loss [29,30,31,32,33,34]. As inflammation resolves, active lesions can evolve into either a chronic active or inactive state [27].

With the advent of DMTs, most patients with chronic MS have far more chronic active and inactive lesions compared to active [27,29,35]. Chronic active lesions, sometimes referred to as mixed/active lesions, are thought to slowly expand and are characterized by a prominent rim of activated, ameboid, and lipid-rich myeloid cells surrounding the lesion border [35,36,37,38]. The core of chronic active lesions is devoid of myelin and lacks sufficient populations of oligodendrocyte progenitor cells (OPCs) and OLs to support robust remyelination [27,39]. Likewise, inactive lesions exhibit severe demyelination and are most closely associated with late stages of MS [27,29]. While inactive lesions can arise directly from active lesions, they often form where the myeloid cell rim around chronic active lesions has dissipated, leaving behind a demyelinated core with some surviving axons, sparse myeloid cells, and virtually no surviving OLs or OPCs [27,29,34,35].

In addition to demyelination, both chronic active and inactive MS lesions are characterized by gliotic astrocytes within the lesion core [27]. Despite being among the most abundant cell populations in MS lesions, astrocytes were historically regarded as pathological bystanders, thought to respond only after demyelination [40,41]. Importantly, recent evidence suggests that astrocytes play a critical role in both lesion formation and the resolution of inflammation in MS and its associated animal models [40,42,43,44]. Furthermore, astrocyte-derived biomarkers have emerged as indicators of disease progression in MS. In particular, elevated glial fibrillary acidic protein (GFAP) levels in serum and cerebrospinal fluid correlate with chronic lesion burden, disability scores, and are also associated with PIRA [45,46,47,48,49]. Given the neurodegenerative pathology of chronic active and inactive lesions, there has been growing interest in astrocyte-directed therapeutic strategies aimed at suppressing pathogenic signaling while preserving neuroprotective functions [50,51]. As a result, a deeper understanding of the role of astrocytes in MS pathophysiology is needed to identify novel therapeutic targets to limit neurodegeneration and disease progression. In this review, we discuss the multifaceted roles of astrocytes in MS and its animal models, with a particular focus on emerging signaling pathways and the evolving concept that lesion-core astrocytes actively regulate neuroinflammation and chronic lesion biology.

A focused literature search was performed to identify studies relevant to astrocyte biology in MS and its animal models, with particular emphasis on astrocyte heterogeneity, inflammatory and neuroprotective functions, immune and transcriptional regulation, and MS lesion pathology. Relevant publications were identified through searches of PubMed/Medline and supplemented by review of reference lists from key articles. Studies were selected based on their contribution to the mechanistic understanding of astrocyte function in CNS inflammation, demyelination, and disease progression.

## 2. Astrocytes

### 2.1. Astrocyte Morphology

**Figure 1:**
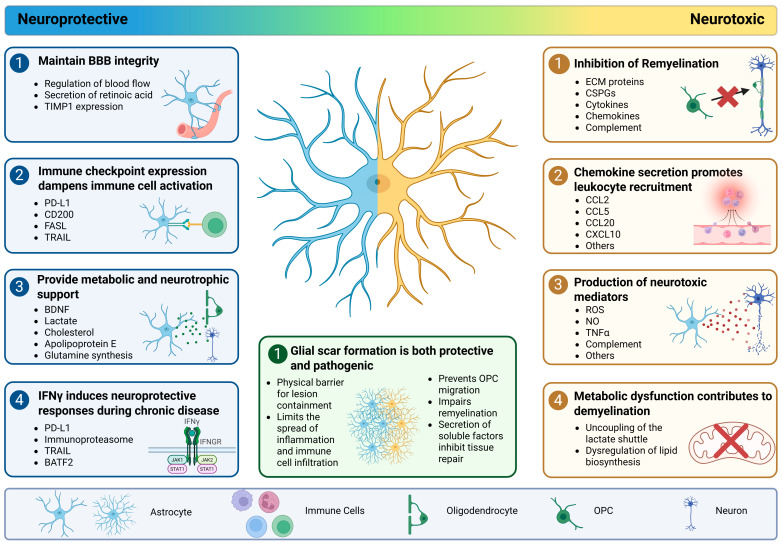
Spectrum of Astrocyte Function during MS. Astrocytes can exert both neuroprotective and neurotoxic functions during MS, with their effects existing along a functional spectrum rather than within defined activation states. Under neuroprotective conditions, astrocytes help maintain BBB integrity, regulate blood flow, and provide metabolic and trophic support to neurons and OLs (brain-derived neurotrophic factor (BDNF), lactate, cholesterol, apolipoprotein E, and glutamine). Astrocytes also limit inflammation by expressing immune checkpoint and interferon-inducible molecules that dampen neuroinflammatory signaling. Conversely, astrocytes can contribute to MS pathology by secreting extracellular matrix components and chondroitin sulfate proteoglycans (CSPGs) that impede OPC recruitment and remyelination, as well as cytokines, chemokines, reactive oxygen and nitrogen species, and complement proteins that drive neuroinflammation. Importantly, glial scar formation, a hallmark of chronic active and inactive MS lesions, represents a functionally complex astrocyte response that can both limit the spread of inflammation and immune cell infiltration and inhibit processes associated with remyelination. Collectively, these diverse functions highlight the context-dependent roles of astrocytes as critical regulators of the lesion microenvironment in MS. Created in BioRender. Williams, J. (2026); https://BioRender.com/yc2akaj (accessed on 10 July 2026).

Among the diverse glial populations in the CNS, astrocytes are one of the most abundant, accounting for approximately 20–40% of all CNS cells [52,53]. Although astrocytes were once viewed primarily as structural support cells, they are now recognized as dynamic participants in CNS function. Beyond maintaining tissue homeostasis, astrocytes regulate neuronal metabolism, shape and refine synaptic connectivity, control extracellular ion and neurotransmitter balance, preserve blood–brain barrier (BBB) integrity, and orchestrate immune signaling [54]. Importantly, astrocyte morphology and function vary considerably across CNS region, reflecting adaptations to the specialized environments in which they reside [55,56] (Figure 1).

Morphologically, astrocytes display a complex, highly branched architecture in which multiple primary processes extend from the cell body and further elaborate into extensive networks of fine terminal processes [55]. Among these extensions, astrocytic endfeet represent a specialized structure that borders blood vessels, positioning astrocytes to sense and regulate vascular signals [57,58]. Astrocytes also extend fine membranous processes, termed leaflets, that closely associate with synapses and facilitate intercellular communication through gap junctions [59].

Astrocytes were historically divided into two broad classes, protoplasmic and fibrous, based primarily on their anatomical distribution and morphological characteristics. However, this binary classification has expanded with the identification of additional specialized astroglial populations, including Müller cells, Bergmann glia, velate astrocytes, and interlaminar astrocytes [60]. Protoplasmic astrocytes are primarily localized within gray matter and are characterized by a highly complex morphology, with numerous fine processes that extensively occupy the surrounding neuropil, form contacts with synapses, and contact nearby blood vessels. In contrast, fibrous astrocytes are enriched in white matter and exhibit a more linear morphology with fewer, longer processes that align with axonal fibers and associate with specialized regions such as the nodes of Ranvier [55,61,62].

Beyond their morphological diversity, astrocytes can also be distinguished by regionally variable patterns of marker expression. Commonly used astrocyte markers, including aldehyde dehydrogenase 1 family member L1 (ALDH1L1) and the glutamate aspartate transporter, GLAST, demonstrate differences in abundance across distinct CNS regions [55,63,64,65]. Additional markers show preferential enrichment within specific astrocyte populations; for example, GFAP expression is more prominent in fibrous astrocytes associated with white matter tracts, whereas S100β is expressed at higher levels in many gray matter astrocytes [55,64,65,66]. Accordingly, the combined assessment of multiple molecular markers is frequently employed to resolve astrocyte heterogeneity across and within CNS regions, underscoring the extensive diversity of astrocyte populations at both molecular and structural levels.

### 2.2. Homeostatic Functions of Astrocytes

The specialized architecture of astrocytes enables them to perform diverse functions that are critical for maintaining CNS homeostasis. Through their extensive processes, astrocytes establish dynamic interactions with a wide range of neighboring cell types, including endothelial cells, neurons, OLs, and microglia. Among these processes, astrocytic endfeet form specialized contacts with the vasculature throughout the CNS. In mice, these vascular associations achieve near-complete coverage by postnatal day 14 [67]. This is critical as astrocytes are required for maintenance of the BBB and regulation of blood flow [68,69]. Consequently, astrocyte-endothelial crosstalk represents a fundamental mechanism through which astrocytes incorporate vascular signals to maintain CNS homeostasis and coordinate the function of surrounding cellular networks.

Beyond their contribution to BBB regulation, astrocytes are indispensable mediators of neuronal development, function, and survival. Through their extensive cellular processes, astrocytes closely interact with synapses and express a broad repertoire of neurotransmitter receptors and transporters that regulate extracellular signaling molecules. For example, astrocytic uptake of glutamate is essential for preventing excitotoxic accumulation and maintaining appropriate neuronal activity [70]. During early CNS development, astrocytes undergo maturation in parallel with periods of active synapse formation. As part of this process, astrocytes produce a variety of factors, including thrombospondins, that promote synapse assembly and maturation [71,72]. Additional molecules secreted by astrocytes include cholesterol, BDNF, glypicans 4 and 6, secreted protein acidic and rich in cysteine, among others, that further shape pre- and post-synaptic function and support synaptic plasticity [73,74,75,76,77].

Astrocytes also serve as critical metabolic partners for neurons by regulating nutrient availability and energy transfer within the CNS (Figure 1). At synaptic sites, astrocytes recycle glutamate by converting it into glutamine, which can subsequently be utilized by neurons for neurotransmitter synthesis. In addition, astrocytes represent a major source of lactate with studies demonstrating that under physiological conditions, up to 85% of glucose consumed by astrocytes can be released as lactate. This metabolite is then transported to neurons through monocarboxylate transporters to support neuronal energy demands [78,79,80].

The metabolic contributions of astrocytes extend beyond neuronal support to include regulation of OL function and myelin formation. Astrocyte-derived lactate can be transferred through gap junctions to OLs, providing an energy source necessary for the metabolic demands of myelination [81]. In addition, astrocytes supply cholesterol to OLs, predominantly in the form of apolipoprotein E, which is released via the ATP-binding cassette subfamily A1 transporter. Given the high lipid requirements of myelin synthesis, disruption of astrocyte lipid metabolism can impair myelin formation and contribute to persistent hypomyelination [82,83].

Astrocytes also maintain essential communication networks with microglia that regulate CNS development, immune surveillance, and tissue homeostasis. As with neurons and OLs, astrocytes release trophic factors that support microglial survival. Specifically, astrocyte-derived colony-stimulating factor 1, transforming growth factor β2, and cholesterol have all been shown to promote microglial viability [84,85,86]. Furthermore, astrocytic production of interleukin (IL)-33 influences microglial-mediated synaptic remodeling by enhancing synaptic engulfment and pruning during development [87,88]. Together, these findings highlight astrocytes as central coordinators of multicellular communication networks within the CNS, integrating neuronal, oligodendroglial, and microglial interactions to support development, synaptic refinement, and maintenance of homeostasis.

### 2.3. Astrocytes During Neuroinflammation and MS

Astrocytes are among the earliest CNS cells to sense inflammatory perturbations and initiate responses that influence the surrounding microenvironment. Rather than adopting a uniform reactive phenotype, astrocyte responses are highly context-dependent and encompass a broad spectrum of functional states. Nevertheless, astrogliosis is commonly associated with increased GFAP expression together with activation of multiple cellular signaling pathways, including Janus kinase/signal transducer and activator of transcription (JAK/STAT), nuclear factor kappa B (NF-κB), and mitogen-activated protein kinase (MAPK) [40,89]. Reactive astrocyte responses are elicited by a broad range of pathological conditions and are shaped by diverse extracellular signals within the CNS, including cytokines, chemokines, neurotransmitters, growth factors, and pathogen- or damage-associated molecular patterns. One of the hallmark outcomes of astrocyte reactivity is the formation of the glial scar which arises as reactive astrocytes proliferate, undergo morphological remodeling, and accumulate at sites of tissue damage. Although this barrier limits the spread of inflammatory mediators and helps to limit lesion expansion, it may also impede axonal regeneration and tissue repair, highlighting the dual nature of astrogliosis following CNS insult [90,91,92] (Figure 1). Although excessive or persistent astrocyte reactivity can impair tissue repair, mounting evidence indicates that this response is essential for restricting CNS damage, particularly in models of traumatic brain and spinal cord injury. These observations provide important context for understanding the complex contributions of glial scar formation in MS [93,94,95].

Within the spectrum of MS pathology, chronic demyelinating lesions are characterized by the accumulation of reactive astrocytes and the formation of a dense glial scar. This response is particularly evident in chronic active and inactive lesions, where astrocytes establish a cellular barrier that helps confine inflammatory activity and restrict further infiltration of immune cells [40]. Despite this protective function, astrocytic scar formation has long been implicated in disease progression because it can create an environment that is unfavorable for repair. Specifically, the glial scar limits OPC migration into demyelinated lesions while promoting the deposition of extracellular matrix (ECM) molecules and secretion of soluble mediators that inhibit remyelination [96]. Among these, CSPGs deposited at lesion margins interfere with OPC maturation by limiting process extension and differentiation [97,98]. Beyond structural changes, astrocytes can also influence disease progression through the release of inflammatory mediators, such as reactive oxygen species (ROS), nitric oxide (NO), tumor necrosis factor (TNF)-α, IL-6, complement proteins, and chemokines. These factors can directly impair neuronal and OL function while amplifying local inflammatory responses [99,100]. Because astrocytes are positioned at the interface between the CNS parenchyma and the vasculature, their inflammatory outputs can also prominently regulate immune cell trafficking. Astrocyte-derived chemokines including CCL2, CCL5, and CXCL10 promote recruitment of peripheral immune cells into the CNS while production of TNF and vascular endothelial growth factor can stimulate endothelial activation through increased expression of adhesion molecules such as vascular cell adhesion molecule 1 and intercellular adhesion molecule 1 [101,102]. In addition to inflammatory signaling, metabolic dysfunction within astrocytes also contributes to MS pathology by disrupting lactate synthesis and transport, thereby limiting metabolic support for neurons and OLs and further exacerbating demyelination and neurodegeneration [103,104] (Figure 1).

Despite their association with inflammatory signaling and lesion-associated pathology, reactive astrocytes do not contribute exclusively to MS pathogenesis, as they also promote tissue protection and repair. This context-dependent functionality is particularly apparent at the BBB where reactive astrocytes can generate retinoic acid, a signaling molecule that suppresses inflammatory responses and limits the recruitment and adhesion of infiltrating immune cells [105]. Astrocytes are also a major source of tissue inhibitor of metalloproteinase 1 (TIMP1), which restricts ECM degradation and supports maintenance of the glial scar and BBB integrity [106,107]. By reinforcing this barrier, astrocytic scar formation can limit the migration of peripheral immune cells into established lesions and prevent the propagation of inflammatory responses into adjacent CNS tissue [92]. Moreover, astrocytes within MS lesions can directly modulate immune activity through expression of inhibitory molecules, such as programmed death ligand 1 (PD-L1) and the membrane glycoprotein CD200, highlighting their capacity to actively regulate local immune responses [108,109] (Figure 1).

### 2.4. Astrocytes in Experimental Autoimmune Encephalomyelitis (EAE)

Astrocyte responses observed in MS are also recapitulated in EAE, a commonly employed preclinical model for investigating mechanisms of neuroinflammation and demyelination. Although EAE provides valuable insight into immune-mediated CNS injury, it does not capture the full complexity of human MS, including the extent and distribution of inflammatory pathology observed in the human brain. However, variation in EAE induction paradigms, including the use of distinct myelin antigens and mouse strains, enables modeling of diverse disease courses that parallel aspects of acute monophasic, relapsing–remitting, and chronic MS. Across these models, many pathological features shared with MS are observed, providing important evidence for the contribution of astrocytes to disease progression and resolution [110]. Importantly, EAE prominently models spinal cord pathology, a clinically relevant feature of MS given that spinal cord lesions occur in approximately 75–90% of patients and are strongly linked to neurological disability and cumulative disease burden [111,112,113]. Thus, EAE represents a critical experimental model system for defining the cellular mechanisms that shape spinal cord pathology in MS and enhances the ability to better understand the contribution of astrocytes within this understudied CNS region [114].

Astrocyte activation is an early event in EAE pathogenesis, preceding the substantial recruitment of peripheral immune cells into the CNS. Evidence of this early response is demonstrated by increased GFAP expression, which can be detected as early as 3 days following immunization and remains elevated throughout chronic disease stages [115]. The functional contribution of astrocytes during EAE was initially revealed through targeted depletion approaches that examined the consequences of astrocyte loss at distinct stages of disease. In GFAP-HSV-TK mice, selective astrocyte ablation before disease induction using ganciclovir resulted in worsened clinical disease, indicating that astrocytes provide early protective functions during EAE initiation. Conversely, depletion of astrocytes after disease establishment (30 days post-induction) improved clinical outcomes and reduced microglial activation, suggesting that astrocyte functions evolve throughout disease progression [116]. Supporting an early protective role, GFAP-deficient mice also develop more severe EAE accompanied by increased immune cell accumulation within the CNS, further demonstrating the importance of astrocytes in regulating inflammatory responses during disease onset [117].

The effects of astrocyte responses during early EAE are highly dependent on the surrounding inflammatory environment with astrocytes capable of simultaneously engaging both protective and disease-promoting programs. One mechanism through which astrocytes contribute to inflammation is by regulating immune cell recruitment through the production of chemotactic factors. In response to inflammatory cues, astrocytes produce multiple chemokines, including CCL2, CXCL10, and CCL20, that facilitate the migration of peripheral immune cells into the CNS [118,119,120]. The functional importance of these pathways has been demonstrated through astrocyte-specific manipulation of individual chemokines. Loss of astrocytic CCL2 reduced EAE severity, supporting a role for astrocyte-derived CCL2 in promoting inflammatory cell recruitment [121]. Similarly, astrocyte-specific deletion of CXCL10 delays disease onset, although it does not fully prevent axonal damage, suggesting that additional inflammatory pathways contribute to tissue injury [119]. While the direct contribution of astrocyte-derived CCL20 remains to be determined, studies targeting its receptor CCR6 have shown reduced EAE severity, implicating the CCL20-CCR6 axis as a potential mediator of astrocyte-driven inflammation [122,123] (Figure 1).

Beyond chemokine-mediated immune cell recruitment, astrocytes engage additional inflammatory signaling pathways that influence EAE progression. One such pathway involves the sphingosine-1-phosphate (S1P) receptors, which represent a key therapeutic target of the MS treatment fingolimod. Although fingolimod was developed based on efficacy in EAE and is best characterized for its ability to restrict lymphocyte egress from lymphoid tissues and subsequent CNS infiltration [109,110], evidence suggests that its effects extend beyond peripheral immune cells. Notably, S1P receptor expression is increased in astrocytes within and surrounding MS lesions [40]. Consistent with a functional contribution of astrocytic S1P signaling, selective deletion of S1P receptors in astrocytes diminishes the therapeutic benefit of fingolimod in EAE, revealing an important role for astrocytes in mediating treatment responses [124].

Despite their ability to promote inflammatory responses under certain conditions, astrocytes also engage protective programs that limit immune-mediated damage and support CNS recovery during EAE. A key example is signaling through the glycoprotein gp130, a shared receptor component for IL-6 family cytokines that activates downstream STAT1/3 and MAPK pathways. Conditional deletion of gp130 in astrocytes using a GFAP-Cre driver exacerbated EAE severity, demonstrating an important role for astrocyte-mediated gp130 signaling. Mechanistically, this benefit was attributed in part to MAPK signaling, particularly the SHP2/Ras/ERK cascade, which limited immune cell entry into the CNS [125].

Astrocytes also contribute to immune regulation through expression of inhibitory checkpoint molecules [126]. During EAE, Fas ligand (FasL) localizes to endfeet surrounding CNS blood vessels, where it promotes caspase-dependent elimination of infiltrating T cells [127] (Figure 1). Accordingly, mice lacking astrocytic FasL exhibit worsened disease accompanied by increased accumulation of CNS T lymphocytes, indicating that astrocyte-mediated FasL signaling contributes to the resolution of autoimmune inflammation [128]. In parallel, astrocytes provide trophic support to vulnerable neurons through secretion of factors such as BDNF. Disruption of astrocyte-derived BDNF increases demyelination and axonal injury during EAE without substantially altering peripheral immune responses [129]. Collectively, these findings emphasize that astrocytes are active regulators of EAE outcome, integrating immune control, barrier regulation, and trophic support to preserve CNS integrity during neuroinflammation.

### 2.5. Astrocyte Interferon (IFN)γ Signaling in MS and EAE

The diverse and context-dependent functions of astrocytes during MS and EAE raise an important question: which molecular pathways determine whether astrocytes adopt protective or pathogenic functions? Among the signaling pathways implicated in regulating astrocyte biology, IFNγ has emerged as a particularly intriguing mediator because it has been linked to both disease exacerbation and tissue protection.

Astrocyte responses in MS and EAE are shaped by a complex inflammatory milieu contributed to by infiltrating immune cells. Among the cytokines present in this environment, IFNγ is particularly abundant during the early phases of disease and is produced predominantly by T helper 1 (Th1) and cytotoxic T cells, with additional contributions from natural killer (NK) cells [130,131]. Cellular responses to IFNγ are initiated through engagement of the IFNγ receptor (IFNγR), triggering activation of the JAK1/JAK2-STAT1 signaling cascade. Following receptor activation, STAT1 undergoes phosphorylation by JAK1 and JAK2, dimerization, and nuclear translocation to induce the expression of interferon-stimulated genes (ISGs) [119]. ISGs regulate diverse immune processes, including antigen presentation, chemokine production, and antimicrobial defense [132,133].

Historically, IFNγ has been regarded as a predominantly pro-inflammatory cytokine in MS because it promotes activation of antigen presenting cells (APCs) and facilitates immune cell infiltration into the CNS during disease initiation and acute inflammatory stages [131,134,135,136]. This view was further supported by an early clinical trial in patients with RRMS in which treatment with IFNγ resulted in clinical deterioration in 7 of 18 participants, an affect attributed to enhanced activation of peripheral APCs [137]. However, observations from progressive MS suggest a more complex biological role. Higher circulating concentrations of IFNγ have been associated with improved clinical status in progressive disease, whereas lower serum levels correlated with worse outcomes, raising the possibility that the effects of IFNγ evolve as disease progresses [138].

Studies in EAE have provided compelling evidence that the biological effects of IFNγ extend beyond its well-established pro-inflammatory functions, particularly during the chronic phase of disease. Administration of IFNγ, either intraventricularly or systemically, attenuated clinical disease and reduced mortality in rats with EAE [139]. Likewise, systemic delivery of IFNγ in a chronic-relapsing mouse model delayed subsequent disease relapses, further supporting a protective role during later stages of neuroinflammation [140]. Complementary loss-of-function studies have reached similar conclusions. EAE-resistant mouse strains, including BALB/c mice, became susceptible to disease and exhibited greater disease incidence and severity following neutralization of endogenous IFNγ before immunization [141]. Consistent with these observations, genetic deletion of either IFNγ or the IFNγ receptor resulted in earlier disease onset and more severe clinical disease after EAE induction compared with wild type controls [142,143].

The protective effects of IFNγ during chronic EAE are, at least in part, mediated through direct signaling within astrocytes. Selective impairment of astrocytic IFNγ responses using dominant negative IFNγ receptor expression resulted in increased demyelination at peak disease, persistent neurological deficits, and impaired recovery compared with control animals, demonstrating the importance of astrocyte-intrinsic IFNγ signaling in limiting CNS damage [144]. Mechanistically, IFNγ promotes the expression of several astrocyte-derived protective pathways, including induction of immunoproteasome components that enhanced clearance of polyubiquitinated proteins and reduced cellular oxidative stress. Consistent with this function, pharmacologic inhibition of the immunoproteasome during chronic EAE worsened clinical disease and increased markers of oxidative injury within astrocytes [145].

Beyond regulation of cellular stress responses, astrocytic IFNγ signaling also promoted local immune regulation through induction of inhibitory checkpoint pathways. Astrocytes increased expression of PD-L1 during EAE, and astrocyte-specific deletion of IFNγR1 using an *Aldh1l1*-Cre driver prevented this upregulation, directly linking PD-L1 expression to IFNγ signaling in astrocytes. Functionally, astrocyte-derived PD-L1 promoted immune cell apoptosis in vitro, while activation of PD-1 signaling during chronic EAE reduced CNS leukocyte accumulation and stabilized clinical disease [126]. Moreover, specific anti-inflammatory subsets of astrocytes have also been identified that express both lysosomal-associated membrane protein (LAMP)1 and TNF-related apoptosis-inducing ligand (TRAIL), whose expression is driven by IFNγ secreted from meningeal NK cells and regulated by the gut microbiome. LAMP1^+^TRAIL^+^ astrocytes limited inflammation through induction of T cell apoptosis, conferring clinical protection during EAE [146] (Figure 1). The observation that IFNγ can exert anti-inflammatory and neuroprotective effects in astrocytes despite its well-established pro-inflammatory functions in other cell types raises important questions regarding the downstream signaling pathways that mediate these effects, including transcriptional regulation of inflammatory gene expression.

## 3. Activator Protein 1 (AP-1) Signaling in MS and EAE

**Figure 2:**
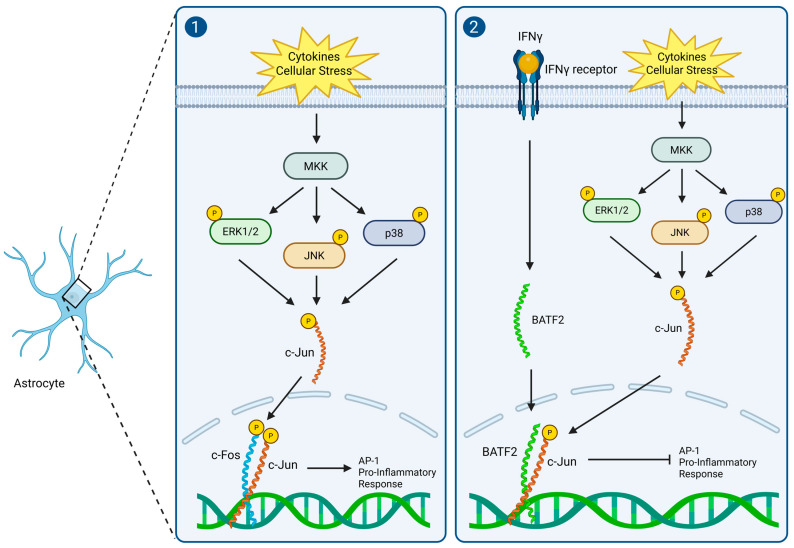
Proposed Model of AP-1 Regulation by BATF2 in Astrocytes. (1) During neuroinflammation, astrocytes are exposed to extracellular signals, including inflammatory cytokines and cellular stressors. In response, MAPK signaling is activated through MAPK kinases (MKKs), resulting in phosphorylation of ERK1/2, JNK, and p38. These pathways promote activation of c-Jun, which can dimerize with c-Fos to form the canonical AP-1 transcription factor complex. AP-1 subsequently binds target promoters that drive the expression of pro-inflammatory genes. (2) IFNγ signaling induces the expression of BATF2, a transcriptional regulator that has been shown to interact with AP-1 family proteins, suppressing their activity. Based on evidence from several peripheral cell types, together with studies demonstrating IFNγ-induced BATF2 expression in astrocytes and BATF2 occupancy at AP-1-associated motifs, BATF2 is proposed to interact with c-Jun at AP-1-regulated loci, thereby limiting AP-1 transcriptional activity and suppressing pro-inflammatory gene expression. Through this mechanism, BATF2 may further contribute to the immunoregulatory and neuroprotective functions of astrocytes during chronic neuroinflammation. Created in BioRender. Williams, J. (2026); https://BioRender.com/y4gsx48 (accessed on 10 July 2026).

Beyond IFNγ-dependent pathways, astrocyte inflammatory responses are regulated by additional transcriptional networks, including AP-1, a central mediator of stimulus-dependent gene expression. AP-1 refers to a diverse family of basic leucine zipper (bZip) transcription factor complexes that form homo- or heterodimers, each containing a DNA-binding basic region and a leucine-zipper motif [147]. Members of the AP-1 family include bZip-containing proteins including Jun, Fos, Maf, and activating transcription factors (ATFs) [148,149]. Among these complexes, the c-Jun/c-Fos heterodimer represents the most extensively characterized AP-1 configuration and was among the first AP-1 complexes identified [147,150,151]. AP-1 activity is closely integrated with MAPK signaling pathways, which regulate the phosphorylation, localization, and transcriptional activity of AP-1 components. In particular, extracellular signal-regulated kinase (ERK), c-Jun N-terminal kinase (JNK), and p38 kinase phosphorylate AP-1 components, facilitating their accumulation in the nucleus and regulation of downstream target genes [152] (Figure 2).

AP-1 regulates a broad spectrum of biological processes, including proliferation, differentiation, cell survival, and stress responses, which has made components of this pathway attractive therapeutic targets for many cancers [153,154]. Beyond these roles, however, AP-1 family members and their upstream regulatory kinases function as important mediators of immune activation and inflammatory gene expression. Evidence of enhanced AP-1 pathway activity has been observed in MS, where increased JNK expression is detected within chronic active lesions [155] and elevated JNK activity is present in peripheral blood mononuclear cells from individuals with RRMS [156]. Consistent with these observations, increased phosphorylated c-Jun has been identified in glial populations within MS lesions, suggesting activation of AP-1 signaling within the CNS during disease [155,157].

Studies in EAE further support the contribution of AP-1 to inflammatory disease progression and indicate that reducing pathway activity can provide protection during neuroinflammation. Pharmacologic targeting of AP-1 components using the c-Fos/c-Jun inhibitor TPN10518 reduced Th1 and Th17 differentiation and attenuated EAE [158]. Similarly, administration of the pan-JNK inhibitor SP600125 limited clinical disease and reduced inflammatory cell accumulation within the CNS [159]. Additional evidence comes from modulation of oxidative stress pathways, as treatment with the copper chelator N-acetylcysteine amide suppressed ROS-dependent activation of JNK and p38, decreased matrix metalloprotease expression, and improved clinical EAE outcomes [160].

Genetic approaches have further revealed that the contribution of JNK signaling to EAE depends on both isoform and cellular context. Loss of JNK1 reduced disease severity, with studies using adaptive transfer models demonstrating that JNK1 expression within non-lymphoid cells is particularly important for disease initiation. Specifically, transfer of JNK1-deficient T cells into wild type recipients was sufficient to induce EAE, whereas wild type T cells failed to induce disease in JNK1-deficient mice, implicating host-derived JNK1 signaling in pathogenesis [161]. In contrast, global loss of JNK2 had limited effects on EAE progression, while JNK3 deficiency modestly exacerbated EAE, highlighting distinct and nonredundant functions among JNK family members [162,163].

Despite the established contribution of AP-1 signaling to neuroinflammation, its specific role within astrocytes remains comparatively understudied in both MS and experimental models of demyelination. Nevertheless, available evidence indicates that astrocytes activate key components of the AP-1/MAPK pathway in response to CNS injury. Immunostaining of chronic active MS lesions revealed elevated expression of both c-Jun and JNK by hypertrophic astrocytes within the lesion compared to adjacent, normal-appearing white matter [155]. Similar findings have been reported in the lysolecithin model of focal demyelination, where endothelin-1 stimulates astrogliosis and GFAP expression within corpus callosum astrocytes through activation of the JNK/c-Jun signaling axis, thereby promoting astrocyte proliferation [164]. Additionally, single-cell RNA sequencing studies in EAE revealed the expansion of distinct astrocyte populations at peak disease that were characterized by pro-inflammatory and neurotoxic gene signatures. Pathway analysis further identified *Jun* among five candidate transcriptional regulators of this subpopulation, suggesting AP-1 pathway involvement in astrocytes during periods of heightened inflammation [165]. In addition to these transcriptional studies, increased expression of phosphorylated ERK, JNK, and p38 has been documented in spinal cord astrocytes of rats at peak EAE, providing further evidence that MAPK-dependent AP-1 signaling is engaged during highly inflammatory states [166].

### Linking IFNγ and AP-1 in Neuroinflammation

The ability of IFNγ to influence AP-1 activity has been demonstrated in several inflammatory settings outside of the CNS. In models of arthritis and peritonitis, IFNγ suppressed the expression of MAPK proteins including ERK, JNK, and p38 [167]. IFNγ is also known to directly inhibit AP-1-induced matrix metalloprotease expression through destabilization of AP-1 proteins [168,169]. However, whether similar regulatory mechanisms operate within the CNS remains largely unresolved, particularly in astrocytes, which represent a major IFNγ-responsive cell population during neuroinflammation. Among the molecules implicated downstream of IFNγ signaling in astrocytes, basic leucine zipper ATF-like transcription factor (BATF)2 is of particular interest due to its ability to interact with and inhibit AP-1 family signaling molecules (Figure 2). The BATF family consists of BATF, BATF2, and BATF3, which share conserved structural features, including a bZip domain and DNA-binding region. Unlike classical AP-1 proteins such as Jun and Fos, BATF and BATF3 lack a transactivation domain [170,171]. In contrast, BATF2 shares some structural similarities with Fos, including an extended carboxy-terminal region, although the functional significance of this domain remains unclear [171].

Functionally, BATF family members interact with Jun proteins and were initially characterized as inhibitors of canonical AP-1 activity [172,173,174]. While BATF and BATF3 are predominantly expressed in hematopoietic compartments and contribute to immune cell differentiation and specialization [171,175,176,177,178], BATF2 exhibits a broader expression profile across tissues [171,179,180]. Compared with the other BATF family members, BATF2 remains less extensively characterized but has emerged as an important regulator of inflammatory responses. Studies to date have primarily focused on BATF2 function in myeloid populations, particularly macrophages and dendritic cells, where its expression is induced by IFNβ, IFNγ, lipopolysaccharide, and other Toll-like receptor ligands. In these settings, BATF2 promotes inflammatory programs and has been implicated in host responses to pathogens such as *Mycobacterium tuberculosis* [171,181,182,183].

**Figure 3:**
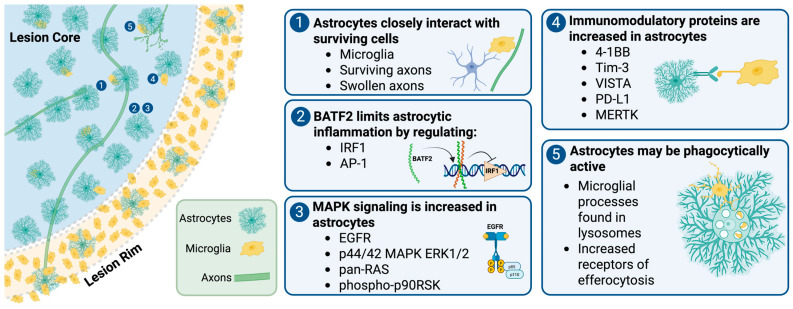
Emerging Astrocyte Functions in the Lesion Core. Chronic active MS lesions contain a demyelinated lesion core populated by gliotic astrocytes, sparse microglia, and surviving axons, surrounded by a rim enriched in activated myeloid cells. Recent spatial profiling studies suggest that astrocytes within the lesion core exhibit distinct molecular signatures indicative of active participation in shaping the local microenvironment. (1) Specifically, astrocytes in the lesion core closely interact with surviving axons and remaining microglia. (2) Lesion-core astrocytes also express elevated levels of BATF2, a transcriptional regulator that is proposed to limit inflammatory signaling through modulation of IRF1- and AP-1-dependent pathways. (3–4) Further, proteins of several signaling networks including MAPK and immune checkpoints are expressed by astrocytes that suggest engagement of pathways associated with cellular survival, proliferation, and immune regulation. (5) Additionally, lesion core astrocytes display evidence of phagocytic activity, including the uptake of microglial debris and increased expression of proteins associated with efferocytosis. Together, these findings suggest astrocytes in the chronic active lesion core dynamically regulate inflammation and contribute to debris clearance, promoting an environment conducive to tissue repair. Created in BioRender. Williams, J. (2026); https://BioRender.com/h3ik9ut (accessed on 10 July 2026).

In contrast to its inflammatory functions described in peripheral immune cell populations, BATF2 appears to serve a protective role in astrocytes during CNS inflammation. IFNγ-dependent induction of BATF2 has been observed in astrocytes, and disruption of this pathway resulted in worsened CNS autoimmune neuroinflammation. Specifically, global loss of *Batf2* led to exacerbated EAE clinical severity, demyelination, and immune cell infiltration. Further, astrocytes deficient in BATF2 also exhibited increased sensitivity to IFNγ and upregulated several interferon-specific proteins including interferon regulatory factor (IRF)1 and caspase-1, which enhanced inflammation in vivo [184] (Figure 3).

Mechanistic studies further suggest that BATF2 may regulate astrocyte transcriptional responses through interactions with AP-1-associated regulatory elements. Chromatin immunoprecipitation sequencing of primary human astrocytes demonstrated BATF2 binding to chromatin at AP-1-associated motifs downstream of IFNγ (Figure 2). In addition, under basal conditions, BATF2 associated with AP-1 target genes involved in cell cycle regulation, including *CCND1* (Cyclin D1), suggesting that BATF2 may regulate a broader range of AP-1-dependent processes beyond inflammatory gene expression [184,185]. Collectively, these data support a model in which IFNγ-induced BATF2 functions as a regulator of AP-1 activity in astrocytes, shaping inflammatory and homeostatic responses (Figure 2).

However, several important questions remain regarding the precise molecular mechanisms underlying this proposed pathway. Current evidence directly linking BATF2 to astrocyte-specific AP-1 regulation remains limited. The protective effects of astrocytic BATF2, IFNγ-dependent BATF2 induction, and BATF2 binding to AP-1-associated motifs are primarily derived from studies demonstrating an anti-inflammatory and neuroprotective role for BATF2 in astrocytes [184]. In contrast, much of the mechanistic framework for BATF2 interactions with c-Jun and AP-1 inhibition comes from peripheral immune cells, where BATF2 can promote pro-inflammatory responses [182,183,186]. Thus, while a direct interaction between BATF2 and AP-1 has yet to be established in astrocytes, existing data support a compelling model in which BATF2 serves as an IFNγ responsive modulator of AP-1-dependent inflammatory signaling and identify this pathway as an important area for future investigation.

## 4. Emerging Signaling Mechanisms in Chronic Active Lesion Core Astrocytes

Chronic active lesions represent a hallmark of progressive MS and are strongly associated with ongoing tissue injury, axonal degeneration, and clinical disability. Although inflammatory activity at the lesion edge has traditionally been viewed as the primary driver of lesion expansion, emerging spatial analyses suggest that astrocytes within the lesion core are not merely passive remnants of tissue damage. Instead, these cells exhibit distinct molecular programs associated with immune regulation, survival, proliferation, and debris clearance, suggesting that core astrocytes may actively impact the health of remaining axons and significantly influence disease progression and clinical outcome.

Recent spatial transcriptomic and proteomic approaches have highlighted that astrocyte responses within chronic active MS lesions are not uniform but instead vary according to anatomical location within the lesion. Astrocytes at the lesion rim, where immune activity is concentrated, exhibit signatures associated with inflammatory signaling, immune interaction, and pathways involved in leukocyte trafficking. In contrast, astrocytes within the lesion core display a distinct molecular profile enriched for pathways related to cell survival, proliferation, immune regulation, and debris clearance, suggesting a more active role in maintaining tissue integrity within areas of established damage. Astrocytes in the perilesional region appear to represent an intermediate state, exhibiting adaptive responses associated with remodeling and communication with surrounding tissue. Together these spatially defined astrocyte states challenge the traditional view of lesion-associated reactive astrocytes as a single homogeneous population and suggests that astrocyte function within chronic MS lesions is highly dependent on local microenvironmental cues. While these data are still emerging, understanding the mechanisms that establish and maintain these distinct astrocyte programs may provide insight into why some lesions remain chronically active while others transition toward repair.

Within this framework of spatially-defined astrocyte states, BATF2 expression within lesion core astrocytes is particularly intriguing. BATF2 is predominantly expressed by astrocytes within the lesion core of chronic active lesions in SPMS and PPMS patient tissue. Whether BATF2 is similarly expressed in active or inactive lesions remains unknown [184]. The spatial specificity of BATF2 expression within the lesion core is striking given the traditional understanding of core astrocytes to be primarily quiescent, structural cells [40,41] and raises the possibility that they may actively participate in regulating inflammatory processes within chronic MS lesions. Consistent with this, BATF2 expression in lesion core astrocytes may play a critical role in regulating the cellular expansion of astrocytes and facilitation of tissue repair through suppression of inflammatory signaling, as it was shown to colocalize with transcriptional regulators such as IRF1 [184]. Furthermore, astrocytes within MS lesions express varying levels of activated AP-1 family proteins such as c-Jun, which BATF2 is known to suppress, further suggesting an active immunomodulatory role for core astrocytes [155,157,174,180] (Figure 3). Thus, BATF2 expression within lesion core astrocytes may represent an endogenous attempt to restrain chronic inflammatory signaling and promote lesion stabilization. Conversely, insufficient induction or functional impairment of this pathway could contribute to persistent astrocyte activation, sustained inflammatory signaling, and a failure of lesion repair characteristic of progressive MS.

In addition to BATF2, astrocytes in the chronic active lesion core have been further characterized as being functionally and spatially diverse compared to other lesion compartments such as the rim and perilesion. Recent spatial protein and transcriptional profiling studies indicate that lesion core astrocytes express molecules linked to proliferation, survival, immune regulation, and phagocytosis, highlighting a more active role for these cells than was previously recognized [187,188,189]. Specifically, several proteins involved in MAPK signaling, including epidermal growth factor receptor (EGFR) and downstream signaling molecules such as p44/42 MAPK (ERK1/2) and phospho-p90RSK were found to be upregulated in the core compared to other compartments assessed including the lesion rim, perilesion, and normal-appearing white matter [187]. Beyond its role in promoting growth and survival, astrocytic EGFR signaling has been associated with anti-inflammatory function and proper glial scar formation in models of spinal cord injury, suggesting that increased EGFR expression may play a role in limiting excessive inflammatory responses within lesions [190] (Figure 3).

Similarly, several immunomodulatory molecules were upregulated in astrocytes within the lesion core, including classical immune checkpoint proteins such as T-cell immunoglobulin and mucin-domain containing (TIM)-3, V-domain immunoglobulin suppressor of T cell activation (VISTA), and 4-1BB, all of which are known to regulate immune cell activation [191,192]. Although T cells are rarely detected in the lesion core, myeloid cells are known to express these and other immune checkpoint proteins, such as PD-1, and are present within the core, albeit in lower abundance compared to the lesion rim [108,126] (Figure 3). Together, this suggests that rather than functioning solely as targets of and/or responders to inflammatory injury, core astrocytes may actively shape the immune landscape of chronic lesions by engaging inflammatory pathways that restrain neighboring immune cells.

Finally, elevated expression of MER proto-oncogene, tyrosine kinase (MERTK) has been reported in lesion core astrocytes, where it functions canonically as a scavenger receptor mediating efferocytosis and the clearance of apoptotic cells [187,193]. Notably, microglial-derived fragments, including TMEM119, have been detected within lysosomal compartments of astrocytes in the lesion core [187]. Although astrocytes are known to exhibit phagocytic activity, these findings suggest that they may also engulf microglial debris during MS, potentially contributing to the removal of necrotic material to prevent its accumulation within the lesion [42,194] (Figure 3).

## 5. Conclusions and Future Directions

The perception of astrocytes in MS has shifted considerably from that of passive pathological bystanders to dynamic regulators of both neuroinflammatory and neuroprotective responses within the CNS. Notably, astrocytes contribute to numerous processes that shape lesion development and progression, including glial scar formation and regulation of inflammatory signaling pathways [40,41]. Evidence from both MS and EAE demonstrates that astrocytes exhibit a high degree of heterogeneity, with distinct populations capable of promoting either tissue injury or repair depending on cues within the local microenvironment. Understanding the mechanisms that govern these divergent astrocyte states may be critical for developing therapies that not only suppress inflammation but also promote tissue repair and limit disease progression in MS.

A central theme emerging from astrocyte biology is that their functions during neuroinflammation are highly context-dependent and dictated by extracellular cues within the local microenvironment. Among these, IFNγ signaling has emerged as a key driver of neuroprotective astrocyte responses, acting through transcriptional programs that limit inflammatory signaling and support CNS homeostasis during chronic disease [126,143,144,184,195]. Specifically, the identification of BATF2 as a downstream regulator of IFNγ signaling highlights the ability of IFNγ to cross-regulate other signaling pathways, including AP-1 and MAPK, which promote pro-inflammatory astrocyte responses such as gliosis and chemokine production [153,164,184]. While a direct connection between IFNγ-induced BATF2 expression and AP-1 regulation in astrocytes requires further elucidation, the convergence of these pathways emphasizes the complexity and integration of signaling networks that govern astrocyte phenotype during neuroinflammation.

A major advance in the field is the recognition that lesion core astrocytes are not merely passive structural cells, but instead represent a dynamic and functionally diverse population, with transcriptional and spatial proteomic signatures converging to highlight roles in immune regulation, survival signaling, and phagocytic activity [187,189]. These findings suggest that astrocytes within chronic active lesions may actively participate in limiting excessive inflammation and preservation of tissue within the core. Given these advances, future work is necessary to expand upon the functional heterogeneity of astrocyte populations across active, chronic active, and inactive lesions, as well as to determine how these populations interact with other cells within the lesion microenvironment. Ultimately, a more comprehensive understanding of astrocyte function and signaling networks in MS lesions has the potential to facilitate the development of targeted therapeutic strategies for patients with chronic, progressive disease.

## Data Availability

No new data were created or analyzed in this study. Data sharing is not applicable to this article.
